# Magnitude and Determinants of Dry Eye Syndrome Among Persons With Diabetes Mellitus

**DOI:** 10.7759/cureus.91685

**Published:** 2025-09-05

**Authors:** Leema Alhussayen, Amna M Alshenqeti, Raghad Alfaqi, Waseem A Aalam

**Affiliations:** 1 Internal Medicine, King Salman Bin Abdulaziz Medical City, Madinah, SAU; 2 Family Medicine, Prince Sultan Military Medical City, Riyadh, SAU; 3 Department of Infectious Diseases, King Abdulaziz Medical City Riyadh, Jeddah, SAU; 4 Ophthalmology, University of Jeddah, Jeddah, SAU

**Keywords:** diabetes mellitus, dry eye disease, dry eye syndrome, glycaemic control, ocular surface disease index

## Abstract

Purpose: To present the prevalence and determinants of dry eye syndrome (DES) among adult Saudi persons with diabetes at a diabetes centre in central Saudi Arabia.

Method: This cross-sectional study included diabetic patients attending a diabetic centre in 2018. The status of DES was based on the Ocular Surface Disease Index (OSDI) questionnaire. The prevalence of DES among patients with diabetes was estimated. The demographic and diabetes status were correlated with the OSDI score.

Results: The DES was found in 237 of 276 persons with a prevalence of 85.9% (95% Confidence Interval 81.8; 90.0). The median OSDI score was 37.5 (Interquartile range (IQR) 20.8; 50). The median hemoglobin A1c (HbA1c) level was 8.6 Mmol/mol (IQR, 7.7-9.7). The OSDI score was not significantly correlated with HbA1C level (Spearman r = 0.794). Age (P = 0.42), gender (P = 0.81), duration of diabetes (P = 0.1), smoking (P = 0.5), contact lens usage (P = 0.36), refractive surgery (P = 0.17), and allergy (P = 0.42) were not significantly associated with OSDI score. Those using insulin for diabetes had a significantly higher OSDI score than those using a type of hypoglycemic medication (P = 0.029). The coverage for care for DES among persons with diabetes was 32.1% only.

Conclusion: The prevalence of DES was high among persons with diabetes. DES was not correlated with duration of diabetes, age, or other known risk factors for DES. Diabetic insulin users diabetic patients had a higher grade of DES compared to those controlling diabetes with oral hypoglycemic medications. Eye care for individuals with diabetes and DES is recommended.

## Introduction

The world is facing a global epidemic of diabetes; 420 million people were suffering from diabetes in 2020, and this is likely to rise to half a billion by the end of this century. The World Health Organization, therefore, recommends supporting people living with diabetes [1]. Nearly one in 12 of the Saudi population aged over 15 years suffers from diabetes. In more than 60 years, almost half of the population had diabetes, whereas around 40% of those aged 45 to 60 years old had diabetes [2]. The Global Eye Health Beyond 2020 report noted impaired vision among persons with diabetes as one of the key factors to be addressed in achieving Universal Eye Care [3]. The most common microvascular complications of diabetes include nephropathy and retinopathy. The ocular complications of diabetes include diabetic retinopathy, cataract, glaucoma, conjunctivitis, corneal pathologies, optic neuropathy, uveal diseases, and dry eye [4]. Type 2 diabetes mellitus is a risk factor for DES since it also alters corneal pathophysiology and causes neuropathy [5]. The risk of and severity of DES were significantly higher in persons with diabetes compared to healthy adults [6]. Poor glycaemic control and a longer duration of diabetes were positively associated with DES in a study by Singh et al. [7]. The presence of DES also negatively impacts the quality of life of a person with diabetes [8]. Therefore, it is crucial to understand the status of DES and the factors that could influence its presence, as well as the severity of DES among individuals with diabetes.

In Saudi Arabia, Helayel et al. noted that the prevalence of DES was 75.2% among the adult Saudi population, and older age, male gender, presence of diabetes, smoking, allergy, autoimmune diseases, contact lens users, and those who had undergone refractive surgery had a significantly higher risk of DES [9]. In a national-level survey of 389 registered diabetics at eight primary Health Centres in 2022, Almohammed et al. reported that a 51.7% prevalence of DES among persons aged 20 years and older with diabetes and poor glycaemic control, but not duration of diabetes, was associated with DES [10]. 

We present the prevalence of patient-perceived severity of dry eye disease in 2018 and its determinants, including glycemic control among diabetes patients attending a diabetes clinic in Madinah, Saudi Arabia.

## Materials and methods

The Ethics and Research Committee of the General Directorate of Health Affairs in Madinah approved this study. Informed written consent was obtained from participants. The tenets of the Helsinki Declarations were strictly adhered to at different stages of this study. The personal identity of the participants was delinked from the data before analysis. The diabetes treatment was administered to all participants, regardless of their agreement to participate in the study.

The individuals with diabetes attending the diabetes clinic for their care during March and April 2018 comprised the study population. Individuals aged 20 years and older who consented to participate were included. Patients with severe systemic conditions related to diabetes who were unable to attend the diabetes clinic were excluded from the study.

This was a cross-sectional study. To represent 25000 registered diabetics in our institute, we assumed that the prevalence of DES would be 75% [11]. To achieve a 95% confidence interval with 5% acceptable error margin, we need at least 285 persons with diabetes to be assessed for DES status. We used the OpenEpi software to calculate the sample size [12].

One ophthalmologist and three internal medicine physicians served as field investigators. The sociodemographic information of the participants included age, gender, nationality, and smoking status. The information regarding the type of diabetes, duration of diabetes, ongoing medications, history of other systemic ailments, medicines administered, use of contact lenses, history of corneal refractive surgery, history of allergies, and allergen exposure was obtained from the case record. The glycemic level was based on the last HbA1c report in the case record.

The DES status was based on the Ocular Surface Disease Index (OSDI) score. There were five questions related to symptoms, four questions about vision, and three questions about environmental triggers for dry eye. The response to each question ranged from 0 to 4. Zero represents none of the time, 1 represents some of the time, 2 represents half of the time, 3 represents most of the time, and 4 represents all the time [13]. The formula to estimate OSDI score was described by Asiedu et al. as follows: (Sum of the scores of all questions answered x 100)/Total number of questions answered x 100. 

The total OSDI score was further categorized as 0-12.9 as normal, 13-22.9 as mild, 23-32.9 as moderate, and 33-100 as severe grade of DES [14].

Glycemic control was based on the HbA1c level noted during the diabetes check-up on the day of the visit. It was further graded as normal (<6.5%), moderate (6.5-9%), and high (>9.0%) [15].

We used the OSDI questionnaire in the Arabic language. A reverse translation was conducted to ensure the quality of the questionnaire was maintained (Appendix 1).

We used internationally accepted scoring for DES and glycemic control. The information from case records, laboratory investigations, and pharmacy records of medication dispensing was reviewed to ensure the reliability of the data. A pilot was held to ensure uniformity of methodology applied by all investigators. Cronbach's alpha was 0.803 for responses to all 12 OSDI questions by 20 pilot participants.

The data was collected on a Microsoft Excel (Redmond, WA, USA) spreadsheet from a Google form. After the consistency check, the data were transferred into the spreadsheet of SPSS Statistics version 25 (IBM Corp., Armonk, NY, USA). The univariate analysis was conducted using a nonparametric method. The categorical variables were presented as numbers and percentages. The normally distributed numerical variables were presented as the mean and standard deviation. For non-normal distributions of numerical variables and of small sample subgroups, we presented the median and interquartile range. The outcome variable, OSDI score, was correlated with numerical determinants to estimate Spearman's rho and two-sided P-value. For a categorical variable with two subgroups, we calculated the correlation of the outcome using the Mann-Whitney U test and reported the corresponding p-values. For more than two subgroups, we estimated Kruskal-Wallis Z and P values. A p-value of <0.05 was statistically significant.

## Results

We interviewed 276 individuals with diabetes who attended the diabetes clinic. Their demographic and diabetes status are given in Table 1. Females constituted three-fourths of the participants. Nearly one-fourth were using insulin alone or in combination with other medications for the management of diabetes. The last HbA1C was <6.5% in 13 (4.7%), between 6.5% and 9% in 155 (56.2%), and >9.0% in 108 (39.1%) of persons with diabetes. 

**Table 1:** Profile of persons with diabetes participating in the dry eye syndrome study

Quantitative variable			
Age	Mean	56.3	
	SDV	13.9	
	Minimum ; Maximum	17- 99	
Duration of diabetes	Median	10	
	Interquartile range	15	
	Minimum - Maximum	0.1; 40	
HbA1C	Median	8.4	
	Interquartile range	2	
	Minimum - Maximum	5.0; 14.0	
Qualitative variables		Number	Percentage
Gender	Male	74	26.8
	Female	202	73.2
Smokers	Yes	22	8
	No	254	92
Contact lens users	Yes	9	3.3
	No	267	96.7
History of corneal refractive surgery	Yes	52	18.8
	No	224	81.2
History of allergy	Yes	4	1.4
	No	272	98.6
Systemic disease	Rheumatoid arthritis	37	13.4
	Parkinsonism	4	1.4
Using medications	Yes	244	88.4
	No	32	11.6
Type of medications	Insulin	28	10.1
	Insulin and other	43	15.6
	Oral hypoglycemic medications	56	20.3
	Hypoglycemic + other oral drugs	37	13.4
	Antihypertensive medication	8	2.9
	Other medications	6	2.2

DES was diagnosed in the past in 76 (27.5%) of diabetic patients. They were suffering from DES for a median duration of two years (IQR 1:3).

The total and subgroup OSDI score of all participants is given in Table 2.

**Table 2:** Ocular Surface Disease Index score in subgroups among persons with diabetes

Subgroup	Number	Median	Interquartile range	Minimum	Maximum
Symptoms	271	9	8	0	20
Activities	272	3	5	04	14
Environmental precipitators	274	2	4	0	12
Total	276	35.7	29.2	0	100

**Figure 1:**
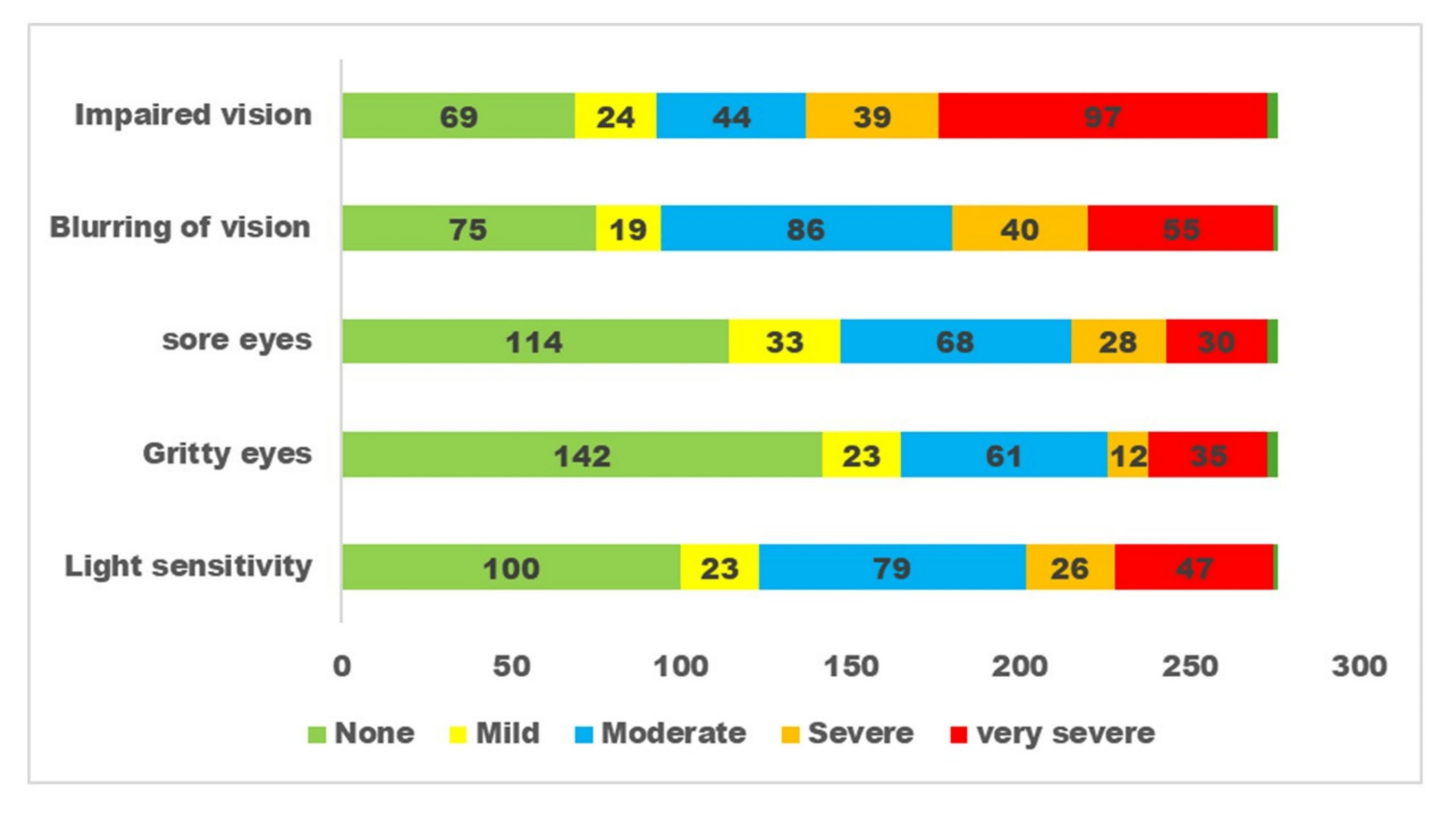
Severity of dry eye disease symptoms perceived by persons with diabetes. The x-axis denotes the number of participants with diabetes. The y-axis denotes different dry eye disease-related symptoms. The green colour denotes persons without symptoms, the yellow colour denotes persons with mild symptoms, the blue colour denotes moderate symptoms, and the red colour denotes severe symptoms.

**Figure 2:**
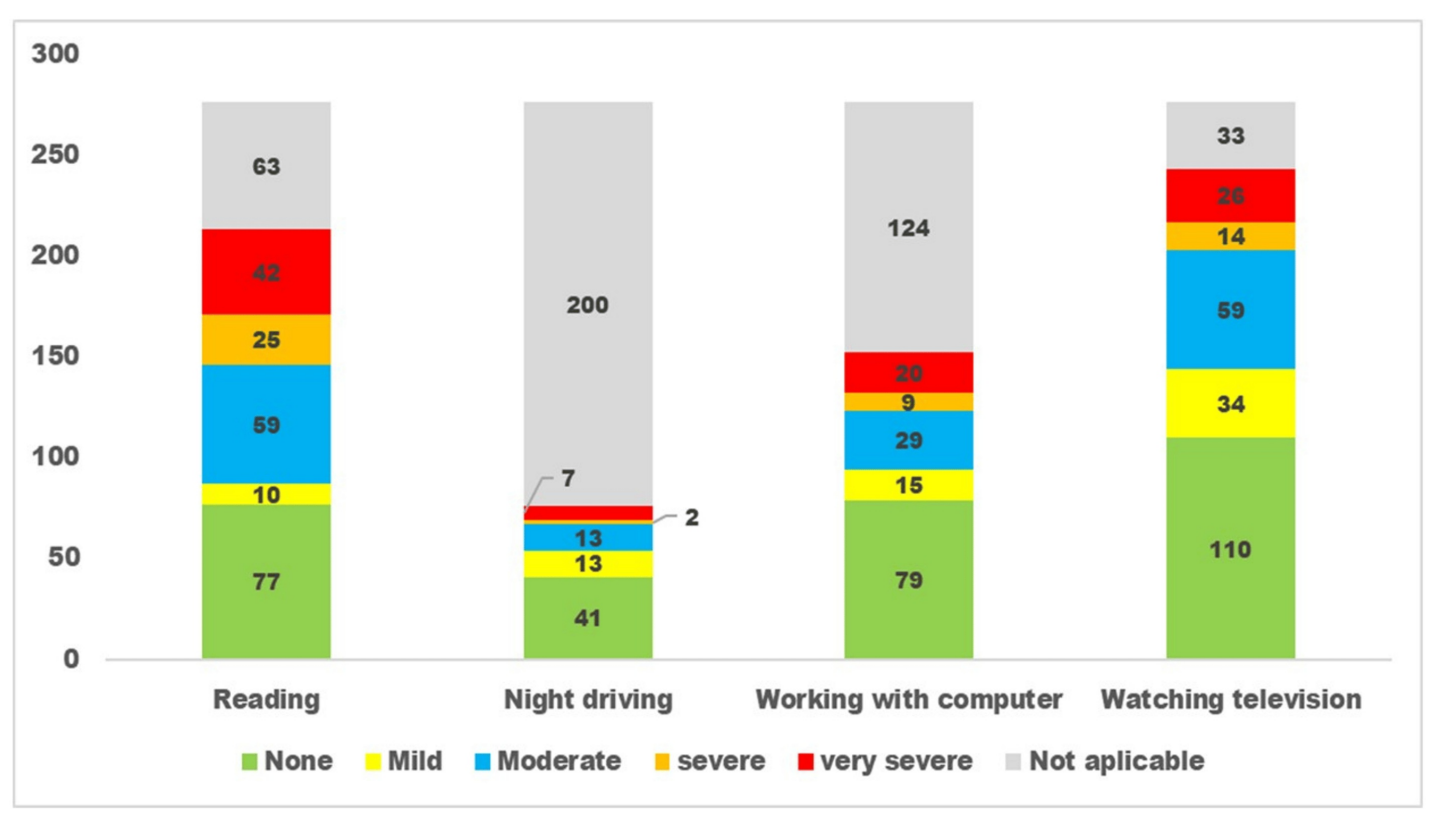
Dry eye disease limiting vision-related daily activities as perceived by persons with diabetes. The x-axis denotes different dry eye disease-related daily living activities. The y-axis denotes the number of participants with diabetes. The green colour denotes persons without limitation, the yellow colour denotes persons with mild limitation, the blue colour denotes moderate restriction, and the red colour denotes severe limitation in vision-related activities.

**Figure 3:**
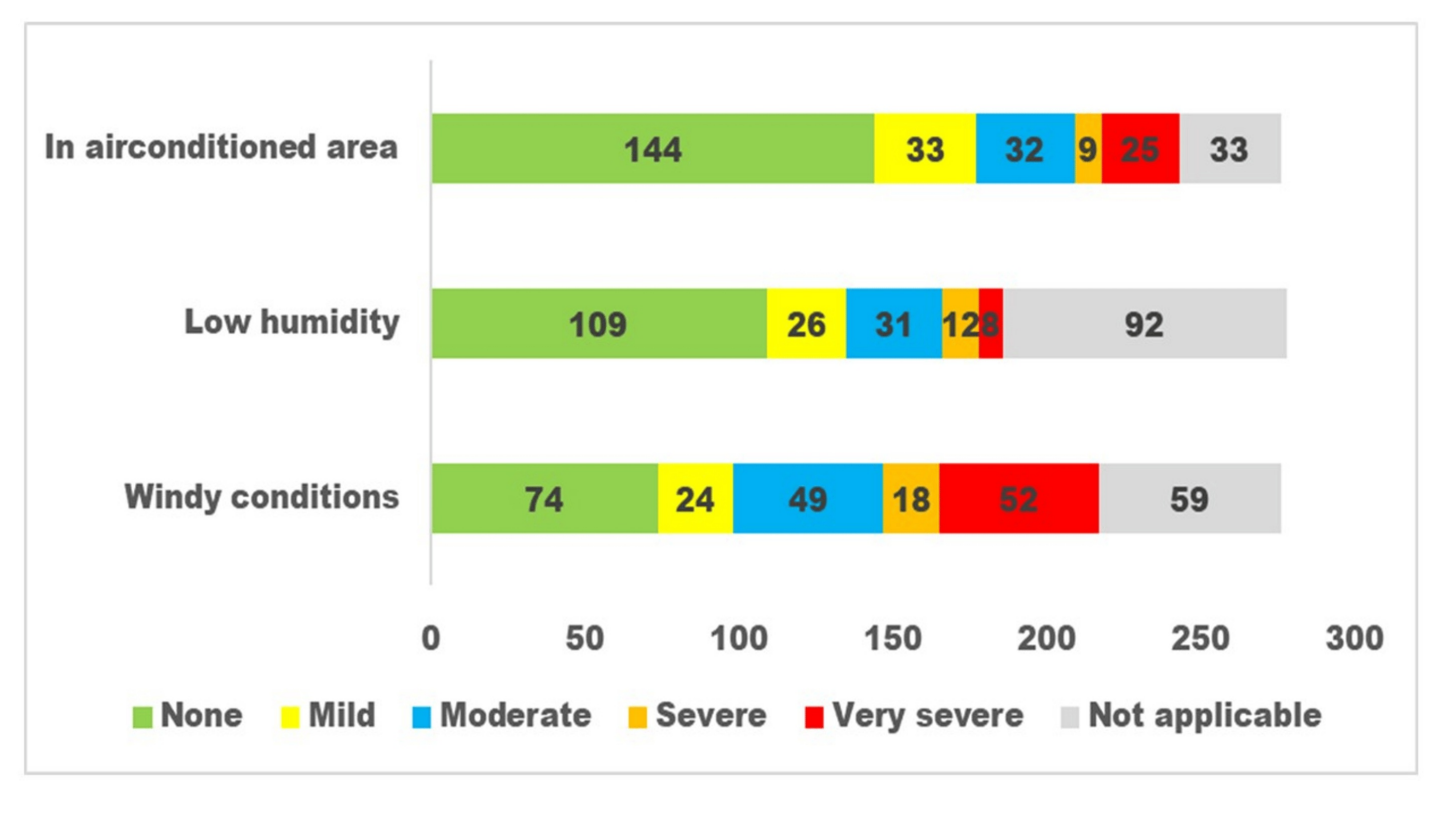
Environmental triggers related to the dry eye disease as perceived by persons with diabetes. The x-axis denotes the number of participants with diabetes. The y-axis denotes environmental triggers. The green colour denotes persons without affection, the yellow colour denotes persons with mild affection, the blue colour denotes moderate affection, and the red colour denotes severe affection on ocular functioning.

The responses to questions related to dry eye disease for the three subgroups are presented in Figures 1, 2, 3.

The prevalence of DES (based on OSDI score) was 85.9% (95% Confidence Interval, 81.8-90.0). Based on the OSDI score, DES was absent in 51 (18.5%), mild in 35 (12.7%), moderate in 42 (15.2%), and severe in 148 (53.6%) of participants. Fifty-five of 146 (37.7%) persons with severe DES were diagnosed and were advised to undergo DES treatment.

We correlated the OSDI score to different variables using univariate analysis (Tables 3, 4).

**Table 3:** Quantitative determinants of dry eye syndrome based on Ocular Surface Disease Index score among persons with diabetes

Quantitative variables	Spearman coefficient	Two-sided P value
Glycemic control (HbA1c level)	-0.016	0.794
Age (years)	-0.009	0.879
Duration of diabetes (years)	-0.093	0.127

**Table 4:** Qualitative determinants of dry eye syndrome based on Ocular Surface Disease Index score among persons with diabetes

Qualitative variables		Number	Median	Interquartile Range	test	P value
Glycemic control	Normal (<6.5%)	13	27.8	29.2	Kruskal wallis	0.25
	Moderate (6.5% - 9%)	155	37.5	29.7		
	Poor (>9.0%)	108	34.1	30.1		
Gender	Male	74	31.3	23.8	Mann-Whitney U test	0.722
	Female	202	37.2	29.4		
Contact Lens user	Yes	9	41.7	35.4	Mann-Whitney U test	0.379
	No	266	35.7	34.8		
Corneal refractive surgery	Yes	52	41.7	25.9	Mann-Whitney U test	0.397
	No	217	34.1	30.3		
Smoking	Yes	22	31.3	33.3	Mann-Whitney U test	0.397
	No	253	35.7	29.2		
Allergy	Yes	4	56.8	41.8	Mann-Whitney U test	0.23
	No	271	35.7	29.2		
Type of main medication	Insulin	66	35.8	27.3	Kruskal wallis	0.028
	Oral hypoglycemic	93	31.3	36.8		
	Other	117	41.7	27.8		

The HbA1c level among persons with diabetes was not significantly correlated with the OSDI score (Spearman P = 0.794). Although the OSDI score of persons with HbA1C of <6.5% was less than that of those with HbA1C >6.5%, it was not statistically significant. The OSDI score among individuals using insulin, oral hypoglycemic medications, and medications for hypertension, arthritis, and other conditions was significant (Kruskal-Wallis P = 0.028). The smokers, contact lens users, old age, and those with corneal refractive surgery were not significantly correlated with DES among persons with diabetes in the present study. Since only one type of medication was used by participants, we were unable to perform multivariate regression analysis to study the interaction of different independent factors and identify the predictors of high OSDI scores.

## Discussion

The prevalence of DES, as determined by the OSDI score, was observed in nine out of 10 individuals with diabetes. The participants had poor control of diabetes, and only one-fourth of them were diagnosed with DES in the past. Eye care was offered to one in four persons with severe DES. Conventional risk factors of DES, like poor glycaemic control, longer duration of diabetes, old age, smoking, contact lens usage, corneal refractive surgery, and arthritis, could not be confirmed in the present study. DES severity differed among individuals using various medications, with higher scores observed among users of oral hypoglycemic agents and other medicines compared to insulin users.

The participants were recruited from a national diabetes centre, and their diabetes status was based on case records, which is more reliable. The internationally recommended questionnaire for OSDI score was used to determine DES status, which is patient-centred and mimics a real-life scenario of an eye problem patient with diabetes experiencing difficulties in daily living. Thus, the study findings, based on an adequate sample, could represent the persons with diabetes managed at the diabetes centre of the kingdom. The undetected cases of DES among persons with diabetes so far are eye-openers for those related to comprehensive eye care for persons with diabetes.

The OSDI-based prevalence of DES was 86% among persons with diabetes in the present study. The symptom-based prevalence of DES was 51.7% among the Saudi diabetic population [10]. Studies in Ethiopia and Brazil revealed 34.8% and 38.3% prevalences among diabetic patients in a hospital [16,17]. When considering the racial differences in prevalence of diabetes and its complications, one should be careful in comparing the prevalence of DES among persons with diabetes of different regions and races [18]. Diabetes is of a much higher proportion among the Arab population compared to the global trend [19]. The prevalence of symptom-based DES among the adult population of the Middle Eastern countries was 28.3%. This review includes six studies held in Saudi Arabia [20]. The high prevalence of DES, noted in the present study, reflects the high rate of diabetes mellitus, improved longevity due to the high quality of health services, and the presence of risk factors for DES in Saudi Arabia [9,21].

The symptom-related questions in OSDI had a higher score than environmental precipitants in our study. Given the dry, hot weather in Saudi Arabia for most of the year, this low score warrants further explanation. Other studies reported higher scores in subgroups related to environmental factors [9,20]. One alternative explanation could be the effect of social desirability bias for symptom-based responses when participants are surveyed at a care-providing institution. The survey time of the year may also matter, as in high temperatures, most participants might be in controlled indoor environments compared to more outdoor activities in winter.

We did not find a correlation between OSDI score and glycaemic level in persons with diabetes. This is in contrast to the findings of Ma et al., who noted a significant correlation between HbA1c and OSDI score [22]. The objective assessment of dry eye disease and its association with poor glycaemic control suggested that it is a significant risk factor for DES [23]. Despite most of the participants being on medications for controlling diabetes, very few had normal HbA1c levels. This poor health behaviour results in three-fourths of diabetics having high HbA1c levels, which was also noted by Alramadan et al. [24]. High DES prevalence and inadequate glycaemic control in most participants may result in a non-significant correlation between these two factors.

We noted that the OSDI score varied significantly among those using insulin, oral diabetes medications, and other medications. Other medications mainly included antihypertensives and those for arthritis. Hypertension, rheumatoid arthritis, juvenile arthritis, and Sjögren's disease are known risk factors for DES [25,26]. It is difficult to determine if DES is related to active disease or side effects of medications used to treat the conditions in a cross-sectional study. The present study had a few limitations. Being a cross-sectional survey, the causal association of DES and glycaemic control should be interpreted with caution. Risk factors like diabetic retinopathy and nephropathy had a positive association with DES, but they were not studied [25,27].

Although eye care services are available to Saudi citizens free of cost in both the catchment area and the diabetes hospital (study site), the coverage of eye services in the diagnosis and care of DES noted in the present study is noteworthy. It could be either DES symptoms that are trivial enough to visit eye care professionals or a lack of awareness among diabetic patients. Establishing protocols for periodic eye assessment with a focus on DES for registered diabetics is recommended [28]. The information on DES was symptom-based only. The objective evaluation of ocular surface and tear film status was not performed. This could complement and give more reliable estimates, as well as risk factors [29]. Screen time is a crucial risk factor for DES [30]. This information, if collected, would further enhance the study outcomes for recommendations.

## Conclusions

The prevalence of DES was high among persons with diabetes. Diabetic insulin users diabetic patients had a higher grade of DES compared to those controlling diabetes with oral hypoglycemic medications. DES was not correlated with duration of diabetes, age, or other known risk factors for DES. Eye care for individuals with diabetes and DES is recommended.

## Appendices

**Table 5:** Ocular Surface Disease Index (OSDI) questionnaire Permission for use of the OSDI was granted by AbbVie Inc.

Symptoms	All the time	Most of the time	Half of the time	Some of the time	Never
Photosensitivity					
Gritty sensation in the eyes					
Painful or sore eyes					
Blurred vision					
Poor vision					
Activities when symptoms are felt and their severity					
Reading					
Driving at night					
PC/ATM machine use					
Watching TV					
Environmental conditions precipitate symptoms and their severity.					
Windy					
Low Humidity					
Within the air conditioning area					
